# Sex-Specific Alterations in Cardiac DNA Methylation in Adult Mice by Perinatal Lead Exposure

**DOI:** 10.3390/ijerph18020577

**Published:** 2021-01-12

**Authors:** Laurie K. Svoboda, Kai Wang, Tamara R. Jones, Justin A. Colacino, Maureen A. Sartor, Dana C. Dolinoy

**Affiliations:** 1 Environmental Health Sciences, University of Michigan School of Public Health, Ann Arbor, MI 48109-2029, USA; trmeier@umich.edu (T.R.J.); colacino@umich.edu (J.A.C.); ddolinoy@umich.edu (D.C.D.); 2 Department of Computational Medicine and Bioinformatics, University of Michigan Medical School, Ann Arbor, MI 48109-2216, USA; wangdaha@umich.edu (K.W.); sartorma@umich.edu (M.A.S.); 3 Nutritional Sciences, University of Michigan School of Public Health, Ann Arbor, MI 48109-2029, USA; 4 Department of Biostatistics, University of Michigan School of Public Health, Ann Arbor, MI 48109-2029, USA

**Keywords:** toxicoepigenetics, DNA methylation, Developmental Origins of Health and Disease (DOHaD), cardiovascular disease, sex differences, heavy metals

## Abstract

Environmental factors play an important role in the etiology of cardiovascular diseases. Cardiovascular diseases exhibit marked sexual dimorphism; however, the sex-specific effects of environmental exposures on cardiac health are incompletely understood. Perinatal and adult exposures to the metal lead (Pb) are linked to several adverse cardiovascular outcomes, but the sex-specific effects of this toxicant on the heart have received little attention. Perinatal environmental exposures can lead to disease through disruption of the normal epigenetic programming that occurs during early development. Using a mouse model of human-relevant perinatal environmental exposure, we investigated the effects of exposure to Pb during gestation and lactation on DNA methylation in the hearts of adult offspring mice (*n* = 6 per sex). Two weeks prior to mating, dams were assigned to control or Pb acetate (32 ppm) water, and exposure continued until offspring were weaned at three weeks of age. Enhanced reduced-representation bisulfite sequencing was used to measure DNA methylation in the hearts of offspring at five months of age. Although Pb exposure stopped at three weeks of age, we discovered hundreds of differentially methylated cytosines (DMCs) and regions (DMRs) in males and females at five months of age. DMCs/DMRs and their associated genes were sex-specific, with a small, but statistically significant subset overlapping between sexes. Pathway analysis revealed altered methylation of genes important for cardiac and other tissue development in males, and histone demethylation in females. Together, these data demonstrate that perinatal exposure to Pb induces sex-specific changes in cardiac DNA methylation that are present long after cessation of exposure, and highlight the importance of considering sex in environmental epigenetics and mechanistic toxicology studies.

## 1. Introduction

Environmental exposures that occur very early in life can have long-lasting influences on cardiovascular disease (CVD) risk [1], consistent with the Developmental Origins of Health and Disease (DOHaD) hypothesis. In spite of this, the underlying mechanisms by which they do so are incompletely understood. Developmental environments may influence CVD risk by altering the structure of the heart, including the final number of cells in the heart or density of blood vessels [2]. Exposures may also alter the expression of genes and their protein products critical for normal cardiac function, such as those of the mitochondrial electron transport chain [2]. There are considerable sex differences in the incidence and pathogenesis of CVDs [3]. For example, although men and women are both prone to ischemic heart disease, the pathogenesis differs between sexes, with males more frequently exhibiting obstructive coronary artery disease [3]. In contrast, ischemia in women is more often due to non-obstructive coronary artery disease or microvascular dysfunction. Likewise, women are more likely than men to experience lethal arrhythmias as a result of pharmacologic interventions (antibiotics, antidepressants, antihistamines) or genetic syndromes [3,4]. The molecular basis for these differences is unclear but likely involves genetic, epigenetic, and hormonal factors [3]. How these important sex differences interact with environmental exposures to influence cardiac health has not been investigated. One potential mechanism by which toxicant exposures early in life can lead to CVD is by interfering with the widespread epigenetic and transcriptional programming that drive normal cardiac development [1]. DNA methylation, or the addition of a methyl group to cytosine bases on DNA (5-methylcytosine), plays a critical role in normal development, and disruptions in DNA methylation have been linked to environment-induced disease. The development of the heart is characterized by dynamic changes in DNA methylation [5], and alterations in this epigenetic mark have been identified in a variety of cardiovascular disease states [1,5,6], as well as with toxicant exposures [6,7,8]. Notably, sex differences in epigenetic modifications and transcriptional profiles are present in the embryonic stem cell stage and persist throughout cardiac differentiation and into adulthood [9,10]. Thus, sex-specific alterations in DNA methylation by environmental exposures may have important implications for sexually dimorphic cardiovascular health.

Recent incidents of Pb contamination in municipal water systems underscore the ongoing threat that Pb poses to human health in the US and worldwide. In addition to drinking water, common sources of Pb exposure in the US include household dust from Pb-based paint, imported consumer products, and industrial exposures [11]. Worldwide, e-waste recycling, traditional medicines, industrial emissions, and glazed ceramics comprise additional sources of exposure [11]. It is estimated that 815 million children worldwide have blood lead concentrations of at least 5 μg/dL, a level at which adverse neurodevelopmental, psychiatric and neurological outcomes are well-documented [11,12]. In addition to the established effects on the nervous system, the contribution of Pb to CVD mortality in the US is far greater than previously thought [13]. Pb exposure is linked to high blood pressure, myocardial infarction, stroke, and cardiac arrhythmias in humans and animals [13,14,15,16,17,18]. In a rodent model, neonatal Pb exposure leads to enhanced sensitivity to the arrhythmogenic effects of norepinephrine in adulthood, suggesting that early development is a critical window of susceptibility to the effects of Pb on cardiac health [19,20]. In spite of established links between Pb and CVD, sex differences in these outcomes, and the underlying molecular mechanisms, are unclear. Early life exposure to Pb plays a clear role in reprogramming of DNA methylation in several non-cardiac tissues, and sex-specific effects of perinatal Pb exposure on DNA methylation have been reported [21,22]. However, the sex-specific effects of developmental Pb exposure on the epigenome of the heart, and the implications this may have for disease, are unknown. In this work, we hypothesized that Pb exposure during this critical window of development would also affect cardiac DNA methylation. To test this hypothesis, we utilized a mouse model of perinatal environmental exposure to investigate the effects of an environmentally relevant dose of Pb on DNA methylation in the hearts of adult male and female mice.

## 2. Materials and Methods

### 2.1. Animal Exposure Paradigm

**Figure 1:**
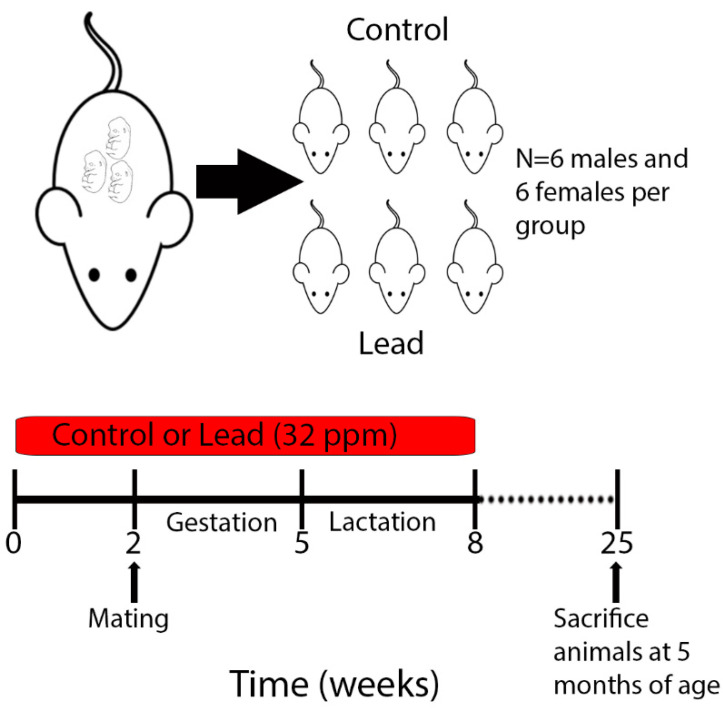
Schematic of experimental design and sample collection. Dams were exposed to Pb 2 weeks prior to mating via drinking water. Maternal (and, in turn, offspring) exposure continued until weaning, when offspring reached 3 weeks of age. 6 males and 6 females per exposure were sacrificed at 5 months of age for tissue collection and ERRBS analysis.

This work was conducted as part of a larger study under the National Institute of Environmental Health Sciences (NIEHS) Toxicant Exposures and Responses by Genomic and Epigenomic Regulators of Transcription (TaRGET II) Consortium [23,24]. Mice utilized for this study have recently been described [8]. Procedures for Pb preparation and exposure were conducted exactly as outlined previously [24]. Briefly, virgin *a/a* females (6–8 weeks old) were mated with virgin *a/a* males (7–9 weeks old), and randomly assigned to receive control or Pb through drinking water. Pb-acetate was mixed with water to result in a Pb concentration of 32 ppm in drinking water, which results in a human-relevant maternal exposure in the 16–60 μg/dL range [22,24]. Pb-supplemented water was made by dissolving Pb (II) acetate trihydrate (Sigma-Aldrich) in a single batch of distilled water, and Pb concentrations were verified using inductively coupled plasma mass spectrometry with a limit of detection of 1.0 µg/L (ICPMS; NSF International, Ann Arbor, MI, USA). Animals were maintained on a phytoestrogen-free modified AIN-93G diet (TD.95092, 7% Corn Oil Diet, (Envigo, Indianapolis, IN USA). Dams were exposed to either control or Pb-supplemented drinking water for two weeks prior to mating, and exposure continued during gestation and lactation. After weaning on postnatal day 21, the resulting pups were weighed and switched to Pb-free drinking water (Figure 1). Approximately 1–2 male and 1–2 female offspring per litter were followed until 5 months of age (*n* = 6 animals per sex/exposure- control female, control male, Pb female, Pb male). All animals had access to food and drinking water *ad libitum* throughout the experiment, remained on a 12-h light/dark cycle, and were housed in polycarbonate-free cages. Health checks were carried out daily by lab personnel and the University of Michigan Unit for Laboratory Animal Medicine (ULAM, Ann Arbor, MI, USA). This study protocol was approved by the University of Michigan Institutional Animal Care and Use Committee (IACUC), protocol # PRO00009800.

### 2.2. Euthanasia and Tissue Collection

Each mouse was weighed on a weekly basis (Mettler Toledo, Columbus, OH, USA) and given regular health checks. Health checks consisted of a general assessment of appearance (fur coat intact and well-groomed, eyes clear, no signs of fight wounds) and behavior (mobility, nest building, etc.). In addition to checks by lab personnel, a designated animal handler from ULAM checked these cues on a daily basis, and a veterinarian assessed the health status of the mice at least once a week. Animals were euthanized at 5 months of age according to protocols established by the TaRGET II Consortium (Figure 1 and [23]). Euthanasia and tissue collection procedures were recently described [8]. Heart samples were immediately snap-frozen in liquid nitrogen and stored at −80 °C until DNA and RNA extraction.

### 2.3. DNA Extraction and Enhanced Reduced Representation Bisulfite Sequencing

DNA extraction (1–2 male and 1–2 female mice per litter) and sequencing were performed as outlined previously [8]. Enhanced reduced representation bisulfite (ERRBS) was performed at the University of Michigan Epigenomics and Advanced Genomics Cores as described previously [24,25], and each sample met the quality control criteria for next generation sequencing. Bisulfite conversion efficiencies for all samples exceeded 99.8% (Supplementary Table S1). The average mapping efficiency was 58.9% (Supplementary Table S1). Single end, 50 nucleotide sequencing was performed on a HiSeq4000 platform (Illumina, San Diego, CA, USA). Libraries were multiplexed and sequenced over 2 lanes. Library sizes (with adapters) ranged from 200–400 bp, and the average sequencing depth was >118 million reads per sample. On average, this method captured 4.8% of genomic CpGs.

### 2.4. Bioinformatics Pipeline, Quality Control, and Differential Methylation Analysis

DNA methylation analysis, including quality control, trimming, alignment, and methylation calling were conducted exactly as outlined previously [8]. Briefly, we removed CpGs with read coverage >1000 or <10. Opposite strand CpGs at the same position were combined via destranding. Sex chromosomes were included in this analysis. We performed differential methylation testing on individual CpG sites (DMCs), and differentially methylated regions (DMRs) were identified in 1000 bp tiles using the same process. In order to be included in the analysis, sufficient sequencing coverage for a minimum of 4 samples from the Pb group and 4 samples from the control group was required. Differentially methylated CpGs and regions were identified exactly as recently outlined [8]. Run was included as a covariate in the model to adjust for batch effects. After obtaining *p*-values, we adjusted for multiple testing using the FDR approach. Sites and regions with FDR < 0.05 and an absolute difference in methylation of >10% were considered significant. To determine the distribution of differentially methylated sites across the genome, we used the *annotatR* R Bioconductor package (v1.5.9) to annotate the CpGs to the mouse mm10 genome ([26] and methods from reference [8]). To determine whether the proportion of differentially methylated cytosines (DMCs) falling into each annotation was significantly different from the total regions tested, we conducted a Chi Square test [8].

### 2.5. Pathway Analysis of Differentially Methylated Regions (DMRs)

Poly-enrich analysis was conducted using all DMRs with a *p*-value of at least 0.1, with the following settings: genesets = GOBP, GOCC, and GOMF; locusdef = 1 kb, min_geneset_size = 15, max_geneset_size = 2000. For GREAT [27] analysis, BED files of DMRs for each sex were uploaded to the GREAT web interface [27] using the mouse mm10 species assembly. Association rule setting “basal plus extension” was utilized with the following parameters: Proximal: 5 kb upstream, 1 kb downstream, plus Distal: up to 1000 kb. STRING network analysis, genes associated with DMRs were analyzed using default parameters: full STRING network and a required interaction score of 0.4.

### 2.6. Gene Expression Analysis

RNA-seq library preparation and sequencing were performed at the University of Michigan Advanced Genomics Core (N = 6 animals per sex, per condition). Library preparation was carried out using the KAPA mRNA Hyper Prep Kit (Roche, Wilmington, MA, USA) with Dual Indexing Adapters following manufacturer instructions. Quantity and quality of the prepared libraries were confirmed with the Agilent 2200 TapeStation (Agilent, Santa Clara, CA, USA). Sequencing of paired-end 50 base pair reads was carried out on the Illumina NovaSeq 6000 (Illumina, San Diego, CA, USA) in the S2 flow cell. Sequenced reads were trimmed via Trim Galore [28], and quality control was assessed with FastQC [29]. STAR was used for the alignment step [30]. Trimming, QC and alignment were all carried out with default parameters. Normalized read counts for Pb-exposed vs. control samples were obtained for each gene using the TMM method of edgeR [31], stratifying by sex. Statistical analysis was conducted as noted in the next section.

### 2.7. Statistical Analysis

For heart weights and gene expression analysis (lead vs. control for each gene of interest in a targeted analysis), animals were stratified by sex, and linear mixed-effects regression was carried out using the *lme4* and *lmerTest* packages in R version 3.6.1 [32]. Litter-specific random effects were included to account for within-litter correlation. Statistical analysis of overlapping genes or sites between groups was conducted using a hypergeometric test [33]. *p*-values and representation factors are reported, where representation factor = the number of overlapping genes divided by the number of genes expected to overlap by chance.

## 3. Results

### 3.1. Litter Parameters and Phenotypic Effects

**Figure 2:**
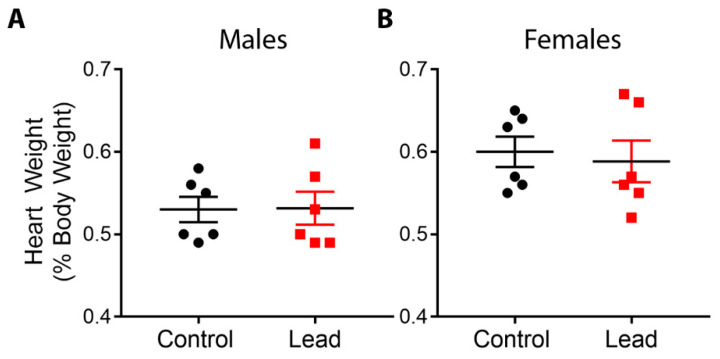
Heart weights, expressed as a percentage of body weight, were assessed for males (**A**) and females (**B**) at 5 months of age. Data were analyzed using linear mixed-effects regression with litter-specific random effects to account for within-litter correlation. Black dots represent control animals and red squares depict Pb-treated animals. There were no statistically significant differences in hearts from Pb-exposed animals compared to controls.

Exposure to Pb during gestation and lactation did not significantly alter litter size, pup mortality, or the percentage of females in each litter, and animal weights at 5 months of age were not significantly different between control and Pb exposed animals [24]. Pb exposure had no significant effect on relative heart weights in either males or females at 5 months of age (Figure 2). This finding may have been due to a relatively small sample size compared to our previous studies in which we observed phenotypic effects of Pb exposure [22,34].

### 3.2. Genome-Wide Changes in DNA Methylation with Developmental Pb Exposure

**Figure 3:**
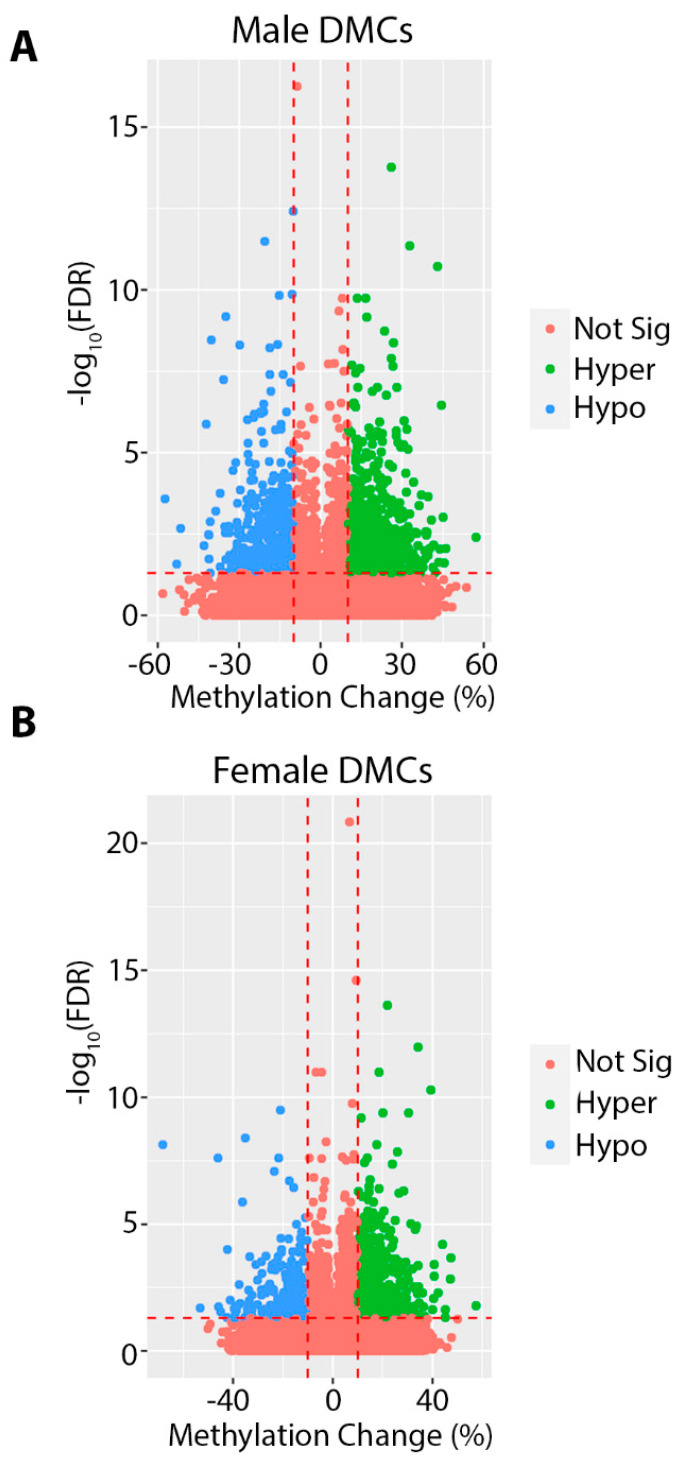
Volcano plots depicting differentially methylated cytosines (DMCs) for Pb exposed compared to control in males (**A**) and females (**B**). DMCs in red did not meet the criteria for significance (at least a 10% change in methylation and FDR < 0.05). Significantly hypomethylated DMCs are shown in blue, and significantly hypermethylated DMCs are shown in green.

**Figure 4:**
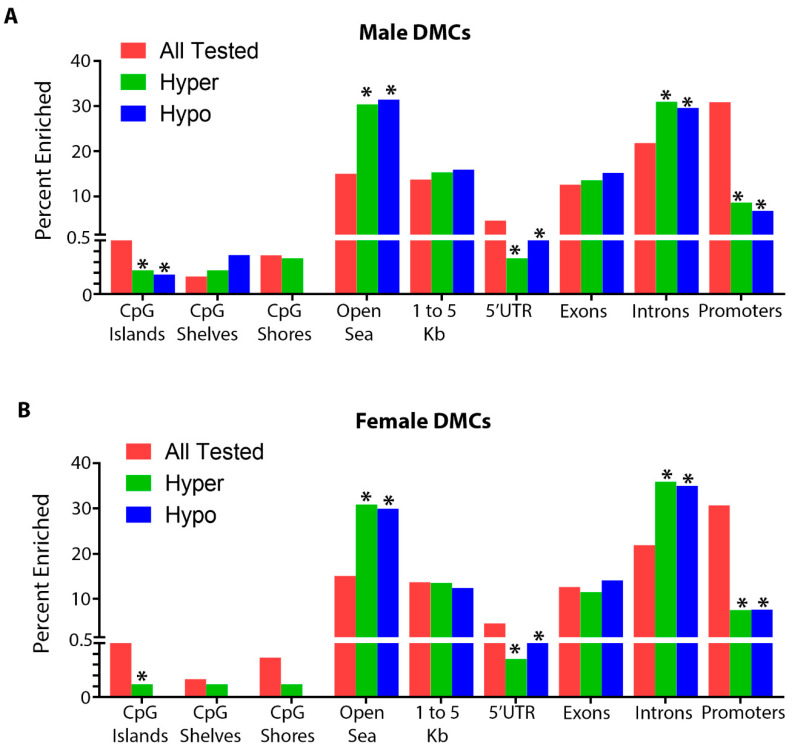
Annotation summary plots depicting the total number of CpGs tested in pink, hypermethylated differentially methylated cytosines (DMCs) in green, and hypomethylated DMCs in blue for each genomic annotation using the R annotatr package for males (**A**) and females (**B**). * Denotes statistically significance, *p* value < 0.05.

In order to investigate the effects of gestational and lactational Pb exposure on DNA methylation, we utilized ERRBS to measure DNA methylation in isolated whole heart tissue from male and female offspring at 5 months of age. Although lead exposure had ceased months before, at 5 months of age we observed >1000 differentially methylated cytosines (DMCs), and several hundred differentially methylated regions (DMRs) in both males and females (Table 1 and Table 2 and Supplementary Tables S2–S5). The total number of cytosines and regions tested was similar between sexes (Table 1 and Table 2). For DMCs, the absolute magnitude of methylation change was as high as 57% in males and 68% in females (Figure 3). For DMRs, we observed a maximum magnitude of 72% and 38% for males and females, respectively (Supplementary Figure S1). We annotated these regions to the mouse mm10 genome and found that, similar to our observations with DEHP and BPA exposed animals [8,35], the majority of DMCs and DMRs fell within open sea and intronic regions, and were depleted from CpG islands and promoters (Figure 4). The top 10 most hyper and hypomethylated DMCs and DMRs, and the genes associated with them, are shown in Table 3 and Table 4.

### 3.3. Pathway Analysis

**Figure 5:**
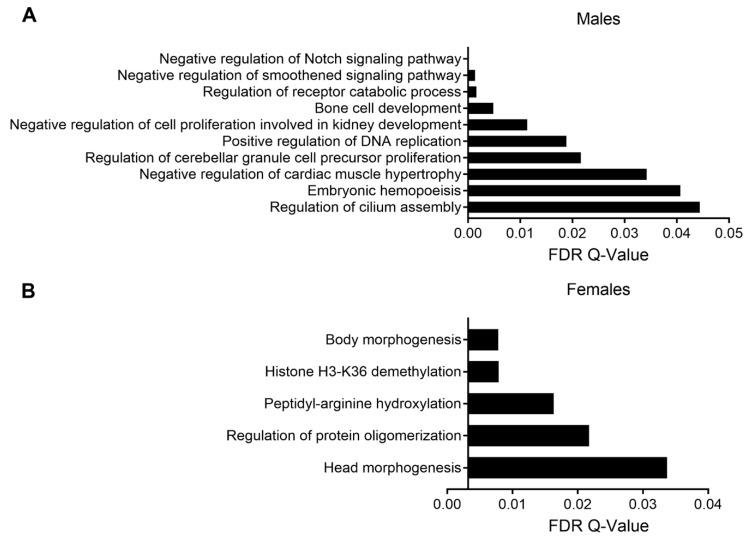
GREAT pathway analysis of DMRs in males (**A**) and females (**B**). Pathways are arranged from most significant (top) to least significant (bottom).

In order to determine the pathways enriched among the DMRs, we first performed analysis using Poly-Enrich [36], which has been shown to have a more accurate false positive rate than other pathway enrichment tests. We stratified our analysis by sex and direction of methylation change. In both males and females, we observed enrichment for pathways relevant to cardiac function, including ion channel activity, but the results were not statistically significant (Supplementary Tables S6–S9). As a parallel approach, we utilized the Genomic Regions Enrichment of Annotations (GREAT) tool [27]. Among males, DMRs were enriched for several pathways important for normal heart development and function, including the Notch and hedgehog signaling pathways (smoothened is a component of the hedgehog pathway) [37,38], as well as regulation of cardiac muscle hypertrophy (Figure 5A). Among females, DMRs were enriched for pathways associated with histone demethylation, arginine hydroxylation, and body morphogenesis (Figure 5B).

**Figure 6:**
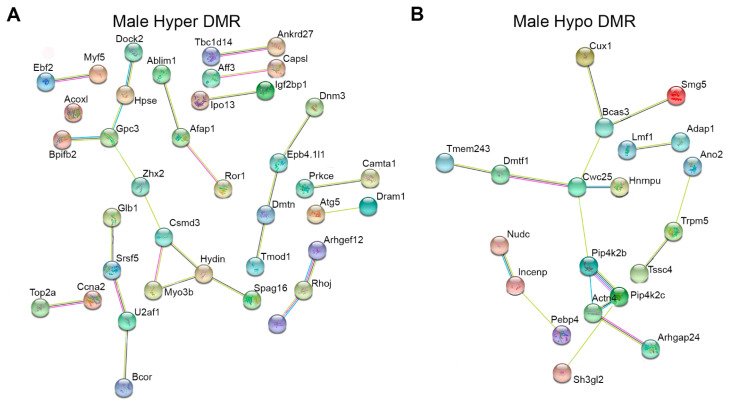
Genes associated with differentially methylated regions (DMRs) in males and females were placed into the STRING network interaction tool. DMR-associated genes in females showed no significant network interactions and are not shown in the figure. Panel (**A**) depicts genes associated with the top 100 most hypermethylated DMRs based on percentage methylation change. Panel (**B**) depicts all hypomethylated DMRs, since there were fewer than 100. To aid in visualization, only the genes that showed significant network interactivity are shown.

In order to further understand whether DMRs interacted within common biological networks, we utilized STRING network analysis. We stratified the data by sex and direction of methylation and conducted separate analyses for each. In both males and females, there were fewer than 100 differentially hypomethylated genes, so we included all of them in the analysis (35 hypo DMRs in females and 67 hypo DMRs in males). For differentially hypermethylated regions in both sexes, we included the top 100 genes with the largest changes in methylation. In females, the interactions among DMR-associated genes were not statistically significant for hyper or hypomethylated genes. In males, however, we identified significantly more interactions than would be expected by chance for both hypo and hypermethylated DMRs (enrichment *p*-values *p* = 0.003 and *p* = 0.009 respectively, Figure 6). Many of the genes in these networks are important for normal heart development and function, or are associated with disease. Among hypermethylated regions, they included *Afap1*, *Prkce*, *Atg5*, and *Tmod1*. Among hypomethylated regions, they included *Bcas3*, *Cux1,* and *Hnrnpu*. Consistent with GREAT analysis, *Atg5* [39,40] and *Cux1* [41,42] interact with the Notch and Hedgehog signaling pathways during normal development and in cancer.

### 3.4. Overlap between Sexes

**Figure 7:**
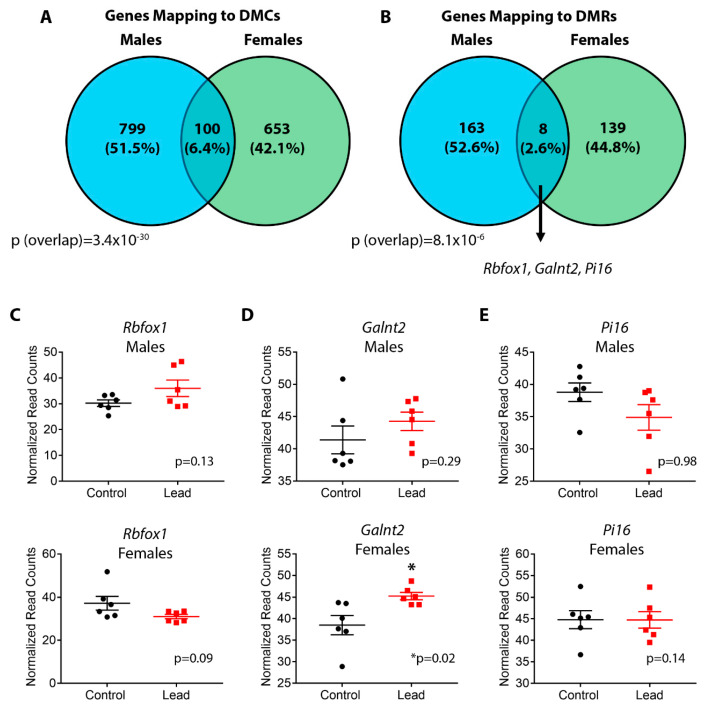
Venn Diagrams showing overlap in differentially methylated cytosine (DMC) (**A**) and differentially methylated regions (DMR) (**B**)-associated genes between males and females. Statistical significance of overlap was determined using a hypergeometric test. In panel B, three DMR-associated genes (Rbfox1, Galnt2, Pi16) that overlap between males and females are highlighted due to their association with cardiovascular disease. Panels (**C–E**) depict normalized RNA-seq read count data for these three genes in males (top panel) and females (bottom panel) at 5 months of age. Black circles and red squares represent control and Pb-treated animals, respectively. Statistical significance was determined using linear mixed-effects regression, with litter-specific random effects to account for within-litter correlation.

We next investigated the sex specificity of DNA methylation after developmental Pb exposure. To this end, we first determined whether any DMCs or DMRs overlapped directly between males and females. Consistent with sex-specific effects on DNA methylation, only two regions were found in common between males and females among both DMCs and DMRs (Supplementary Tables S10 and S11). The sex-specific overlap of DMCs was not statistically significant (*p* = 0.43, representation factor = 1.4), and the overlap of DMRs approached statistical significance (*p* = 0.05, representation factor = 5.4) as calculated by hypergeometric test. We then compared the genes mapping to DMCs and DMRs between males and females to further interrogate sex specificity. In males, DMCs and DMRs mapped to 899 and 171 genes, respectively (Supplementary Tables S2 and S4). In females, we identified 753 and 147 genes associated with DMCs and DMRs, respectively (Supplementary Tables S3 and S5). The vast majority of DMCs and DMRs were sex-specific, with a small subset of genes overlapping between sexes (Figure 7A,B and Supplementary Tables S12 and S13). Although a minority of genes were found to be in common between sexes, the overlaps for DMCs and DMRs were statistically significant (*p* = 3.4 × 10^−30^, representation factor = 3.7 and 8.1 × 10^−6^, representation factor = 8.0, respectively, hypergeometric test). We then determined whether the overlapping genes might represent sex-independent biomarkers of Pb exposure, focusing on the 8 genes in common among DMRs. Of these 8 genes, we identified 3 that were associated with cardiovascular diseases, including *Rbfox1*, *Galnt2*, and *Pi16* [43,44,45]. Altered DNA methylation at each gene occurred in distinct locations based on sex (Table 5 and Supplementary Table S13). Changes in DNA methylation at *Galnt2*, and *Pi16* occurred in the same direction in both sexes (Table 5). Interrogation of the sex-specific DMRs also revealed cardiovascular disease-relevant genes. Among males, these included *Atg5*, *Tmod1*, *Smad6*, *Slc26a6*, *Prkce*, *Ank2*, *Cux1,* and *Lamp2* [46,47,48,49,50,51,52,53]. Among females, they included *Tgfb2*, *Rbfox2*, *Vdr*, *Tlr4*, *Timp3*, *Pde4b*, *Akap1,* and *Grk5* [54,55,56,57,58,59,60,61].

### 3.5. Gene Expression Analysis

We then investigated whether the changes in DNA methylation at sex-independent and sex-dependent DMRs were associated with altered gene expression. To this end, we first interrogated RNA-seq read count data for each of the three sex-independent genes from control and Pb-exposed males and females. RNA utilized in the RNA-seq analysis was extracted from the same tissue samples utilized for ERRBS. Gene expression data for *Rbfox1*, *Galnt2*, and *Pi16* are depicted in Figure 7C–E. *Rbfox1* exhibited trends toward increased and decreased expression in males and females, respectively that did not reach statistical significance (Figure 7C). For *Galnt2*, we observed a significant increase in expression in females but not males (Figure 7D). No significant changes were observed in expression of *Pi16* (Figure 7E). Among cardiovascular disease-relevant, differentially methylated genes in males, *Atg5*, *Ank2*, *Cux1* and *Lamp2* exhibited significant increases in gene expression. Expression of *Tmod1*, *Smad6*, *Slc26a6* and *Prkce* were not significantly different between Pb and control (Supplementary Figure S2A). In females, expression of *Akap1* was significantly increased and *Grk5* was significantly decreased. The remaining genes showed no significant changes in expression with Pb exposure (Supplementary Figure S2B).

## 4. Discussion

Although there are clear sex differences in cardiac physiology and pathophysiology [3], the sex-specific effects of environmental exposures on cardiovascular health are poorly understood. In this work, we demonstrate that Pb exposure during gestation and lactation leads to changes in DNA methylation in the heart that are present in adulthood. The levels of Pb used in this study result in maternal blood Pb levels comparable to those currently observed in women of child-bearing age in poorer countries [62]. Although blood Pb levels have fallen significantly in the US since the 1970s–1980s, levels within this range were not uncommon during the mid-late 20th century [63]. Importantly, children exposed to higher levels of Pb during pregnancy in the mid-late 20th century are now at an age in which CVDs are of significant concern. Our findings are novel for several reasons. To our knowledge, this is the first report demonstrating that Pb exposure during early development leads to altered cardiac epigenetic programming that is present in adulthood. Moreover, sex-specific effects of Pb exposure on the cardiac epigenome have not yet been investigated. Indeed, although the effects of Pb on the nervous system are well-established, this work contributes to a growing body of evidence highlighting potential adverse effects of this metal on the cardiovascular system.

### 4.1. Genome-Wide Changes in DNA Methylation with Developmental Pb Exposure

In this work, we discovered that exposure to Pb during gestation and lactation resulted in genome-wide changes in cardiac tissue DNA methylation. In both males and females, DMCs and DMRs were enriched in introns and intergenic regions of the genome, consistent with what we observed previously for DEHP [8] and BPA [35] exposures. Intergenic and intronic regions of the genome harbor enhancers and other regulatory elements critical for the regulation of tissue-specific gene expression [64]. Likewise, dynamic DNA methylation at these elements is critically important for regulation of normal tissue differentiation, and is de-regulated in the context of diseases such as cancer [65]. Consistent with this, GREAT pathway analysis revealed enrichment of pathways associated with development and epigenetic regulation. In males, this included the regulation of the Notch pathway, as well as smoothened signaling, a component of the hedgehog pathway. Both pathways play critical roles in normal cardiac development, as well as in adult cardiovascular diseases [37,38,66]. In females, these included pathways associated with lysine (H3K36) demethylation and arginine hydroxylation, epigenetic processes that are important for regulation of chromatin structure and function in the heart and other tissues [67,68]. As a complementary approach, we conducted STRING network analysis and found that, in males, genes associated with the DMRs interacted in networks associated with cardiovascular development and disease, including *Afap1*, *Prkce*, *Atg5*, *Tmod1*, *Bcas3*, *Cux1*, and *Hnrnpu* [47,50,52,69,70,71,72]. The implications of altered DNA methylation at these development and disease-relevant loci for long-term cardiac health require further investigation.

### 4.2. Implications of Pb Exposure for Cardiac Function

The effects of Pb-induced changes in DNA methylation on heart function are not yet clear. Pb exists predominately as a divalent cation in the body, and thus interferes with the function of other divalent cations, including calcium. Acute exposure to Pb disrupts cardiac calcium signaling, which is critical for normal heart function, and leads to arrhythmias and impaired cardiac contractility [73,74]. Pb may also exert toxic effects in part through activation of the aryl hydrocarbon receptor [75], and can potentiate the effects of other cardiac toxicants [76]. The effects of developmental Pb exposure on cardiac function, however, are far less clear. In this study, potentially due to the small sample size, we observed no significant changes in heart weights in either males or females, and we did not assess the functional effects of early Pb exposure. Previous work in rats demonstrated that exposure to Pb during the early postnatal period lead to an increased incidence of cardiac arrhythmias in response to norepinephrine in adulthood, and the presence of the toxicant during the early developmental period was necessary for the observed effect [19,20]. These findings suggest that early Pb exposure may affect the sensitivity to additional stressors later in life. Given the extensive epigenetic programming that occurs during this critical window of development, and the observed functional effects during adulthood, it is plausible that developmental Pb exposure may elicit these effects through epigenetic mechanisms. Additional studies designed to investigate this important question are currently underway.

### 4.3. Potential Mechanisms

The molecular mechanism(s) by which Pb exposure leads to altered cardiac DNA methylation are currently unclear. DNA methylation is mediated by DNA methyltransferases (DNMTs), and active removal of this mark occurs via the activity of TET dioxygenases (TETs). DNMTs require the cofactor S-adenosylmethionine (SAM), which provides the methyl group for cytosine methylation. TETs utilize iron (II), ascorbic acid, and alpha ketoglutarate to catalyze the conversion of 5-methylcytosine to the more oxidized products 5-hydroxymethylcytosine (5hmC), 5-fluorocytosine 5 (fC), and 5-carboxylcytosine (5caC). Conversion of these oxidized species back to unmethylated cytosine occurs through thymine DNA glycosylase (TDG)-mediated base excision repair. Thus, Pb may interfere with DNA methylation by altering the expression of these enzymes and/or the levels of their cofactors. Consistent with the first possibility, developmental Pb exposure leads to reduced activity and expression of DNMTs in vitro and in vivo [77,78]. No effects of Pb exposure on expression of TETs or TDG have been reported; however, Pb does cause alterations in the levels of 5hmC [79]. In support of an effect on cofactors, exogenous SAM mitigates the effects of Pb exposure on acute toxicity and adverse neurodevelopmental outcomes, suggesting that Pb may perturb SAM homeostasis [80,81]. Moreover, Pb interferes with the activity of the TCA cycle enzyme isocitrate dehydrogenase, which generates alpha ketoglutarate [16,82]. Vitamin C has also been shown to attenuate the adverse effects of Pb, although whether this is due to modulation of DNA methylation is unclear [83,84].

The molecular underpinnings of sex-specificity in differential DNA methylation are currently unknown. Sex differences in epigenetic profiles are present during cardiac differentiation and in adulthood, highlighting differential epigenetic regulation of cardiac development and function between males and females [9]. During early development, DNA methylation undergoes two distinct waves of widespread erasure and re-methylation in both males and females; however, the kinetics and underlying mechanisms driving this programming differ between sexes. The first wave occurring post-fertilization results in removal of gamete methylation patterns and establishment of the embryonic methylation program [85]. The second wave occurs in the primordial germ cells of the developing embryo, in which sex-specific methylation patterns are established. In pre-implantation embryos, removal of DNA methylation in females occurs via a slower, passive loss of methylation, while in males, demethylation occurs with faster kinetics via an active mechanism mediated by TETs [85]. In the primordial germ cells, the process of re-methylation is complete before birth in males, but in females, this re-methylation continues until puberty [85]. These differences in the timing of methylation erasure and re-establishment may contribute to the observed sex differences in DNA methylation in response to toxicant exposures. Additional mechanistic studies are necessary to address this question.

### 4.4. Sex Differences in the Effects of Pb Exposure on the Heart

In this work, we discovered that the effects of gestational and lactational Pb exposure on the heart are sex-specific. Specifically, we observed few DMCs/DMRs that overlapped directly between males and females, and a minority of DMC/DMR-associated genes were found to be in common between sexes. This is consistent with our previous findings for DEHP exposed heart, as well as lead-exposed liver [8,24]. Genome-wide and site-specific changes in cardiac DNA methylation have been reported for other environmental exposures, but studies thus far have largely focused on males [7,86]. Notably, although the epigenetic mechanisms underlying sex differences in CVD are unclear, sexually dimorphic patterns of DNA methylation are associated with various CVDs [87]. Thus, sex-specific perturbations in DNA methylation might differentially affect susceptibility to adverse cardiovascular outcomes after perinatal exposure to Pb. Among the small number of DMRs that overlapped between sexes, we identified several associated with cardiovascular disease, leading us to hypothesize that these genes may be similarly de-regulated by Pb exposure in both sexes. However, *Galnt2* was the only sex-independent gene investigated that exhibited significant changes in expression and DNA methylation, and altered expression occurred in females but not males. As the locations of the DMRs within each of the genes differed between males and females, differential effects on gene expression are not surprising. Several sex-specific DMR-associated genes also exhibited significant changes in expression concomitant with differential methylation. Collectively, these data suggest that sex had a profound effect on Pb-induced changes in DNA methylation. As only a minority of studies in cardiovascular epigenetics stratify data by sex [88], in spite of clear sex differences in CVD, our work highlights the necessity of considering sex as a biological variable in cardiovascular environmental health studies.

### 4.5. Limitations of the Study

Although our findings have important implications for cardiovascular environmental health, there are several limitations to this study. First, because we did not measure the level of Pb in the heart at 5 months of age, we cannot rule out the possibility that there may still be Pb present in the hearts at this time point. However, the majority of Pb is stored in bone, with a relatively small quantity absorbed into soft tissues [89,90]. Moreover, the turnover of Pb is far more rapid in soft tissues compared to bone [89,90]. Although Pb is released from bone during pregnancy and age-related bone loss, the animals examined in this study did not experience pregnancy or old age [91,92]. Thus, in the absence of Pb exposure for over 4 months, we would expect minimal residual Pb in the hearts of adult animals. Whether Pb remains in the body after early developmental exposure or not, our data suggest that Pb exposure during early life causes epigenetic changes that may have important implications for health across the life course. A second limitation to this study is the use of the ERRBS method, which is designed to enrich for CpG-rich regions of the genome. Because of this bias, it is plausible that other regions of biological significance in the genome were altered by Pb exposure but were not detected using this method. In addition, this method uses a traditional bisulfite conversion protocol, which does not permit us to discriminate between 5mC and 5hmC. Thus, some of the changes in DNA methylation may reflect changes in 5hmC rather than 5mC. Importantly, 5hmC is also present in the heart and is important for cardiac development and disease [93,94]. Studies to investigate the effects of Pb exposure on other epigenetic modifications, including 5hmC, are warranted.

## 5. Conclusions

In conclusion, we have demonstrated, for the first time, that Pb exposure during early development leads to changes in DNA methylation in adulthood that are strongly dependent on sex. This work adds to a growing body of evidence linking the early developmental environment to epigenetic changes in the cardiovascular system. It will be of great interest to determine whether the observed changes in DNA methylation are associated with an increased risk of CVD across the life course.

## Figures and Tables

**Table 1:** Differentially methylated cytosines (DMCs) in 5 month offspring mouse hearts.

Condition	Total	# Hypermethylated (% Total)	# Hypomethylated (% Total)	Total Tested
Female Pb	1204	850 (71%)	354 (29%)	1,229,906
Male Pb	1441	894 (62%)	547 (38%)	1,242,400

# = number.

**Table 2:** Differentially methylated regions (DMRs) in 5 month offspring mouse hearts.

Condition	Total	# Hypermethylated (% Total)	# Hypomethylated (% Total)	Total Tested
Female Pb	243	183 (75%)	60 (25%)	199,245
Male Pb	303	200 (66%)	103 (34%)	200,236

# = number.

**Table 3:** Top 10 differentially hypo- and hyper-methylated cytosines (DMCs) in each sex, ranked by methylation change.

**Males**					
**Chromosome**	**Chromosomal Coordinate**	**Methylation** **Change**	**FDR**	**Gene**	**Genomic Annotation**
7	123,192,720	57.24	0.004	*Tnrc6a*	Exon, Intron
15	27,622,926	46.04	0.009	*Otulin*	Intron
17	45,811,574	45.45	0.024	N/A	Intergenic, Intron
5	113,144,949	45.00	9.49 × 10^−4^	*2900026A02Rik*	Intron
Y	2,408,706	44.97	3.51 × 10^−7^	N/A	Intergenic
5	75,512,841	43.82	0.009	N/A	Intergenic
17	48,467,937	43.36	0.024	*Unc5cl*	Exon, Intron, 3′UTR
17	45,131,087	42.98	1.96 × 10^−11^	N/A	Intergenic
14	74,943,186	42.21	0.048	*Lrch1*	Intron
8	50,297,709	41.54	0.007	N/A	Intergenic, Intron
4	127,676,072	−57.41	2.6 × 10^−4^	N/A	Intergenic
11	101,777,136	−52.95	0.026	*Etv4*	1 to 5 kb, Exon
X	151,886,783	−51.63	0.002	*Huwe1*	Exon
18	58,711,965	−42.84	0.007	N/A	Intergenic
19	46,075,205	−41.21	0.003	*Nolc1*	Promoter
5	15,589,011	−41.20	0.018	N/A	Intergenic, Intron
5	60,922,979	−41.14	0.003	N/A	Intergenic
10	126,913,858	−40.79	0.049	N/A	Intergenic
14	82,306,636	−40.71	0.001	N/A	Intergenic
12	112,184,867	−40.33	3.43 × 10^−9^	N/A	Intergenic
**Females**					
**Chromosome**	**Chromosomal Coordinate**	**Methylation** **Change**	**FDR**	**Gene**	**Genomic Annotation**
11	100,799,435	57.52	0.016	*Stat5b*	Intron
X	93,674,708	47.42	2.16 × 10^−4^	*Pcyt1b*	Promoter, Intron
10	44,097,412	47.17	0.001	*Crybg1*	Intron
11	107,243,282	45.18	0.023	*Pitpnc1*	Intron
10	127,239,429	45.16	0.048	*Kif5a*	Intron
2	153,872,822	43.94	6.39 × 10^−5^	*Bpifb2/Sun5*	1 to 5 kb
5	122,761,313	42.20	0.005	*Camkk2*	Intron
X	138,915,140	40.62	0.001	*Nrk*	Intron
10	49,704,554	40.36	0.008	*Grik2*	Intron
15	97,844,916	40.14	0.023	*Hdac7*	Promoter
19	37,685,435	−68.11	7.33 × 10^−9^	*Cyp26c1*	Promoter
5	119,687,886	−53.30	0.020	*Tbx3os2*	Intron
15	73,723,726	−45.97	2.47 × 10^−8^	*Ptp4a3*	1 to 5 kb, Intron
17	76,107,125	−45.74	0.018	N/A	Intergenic
17	5,507,498	−45.04	0.030	*Zdhhc14*	Intron
12	104,471,048	−42.53	0.047	N/A	1 to 5 kb, CpG Shore
12	104,471,092	−42.19	1.01 × 10^−4^	N/A	1 to 5 kb, CpG Shore
1	70,455,889	−41.23	0.010	*Spag16*	Intron
5	124,782,320	−39.66	0.021	*Dnah10*	Exon
6	101,272,100	−39.34	0.050	*Pdzrn3*	Intron

FDR = False Discovery Rate.

**Table 4:** Top 10 differentially hypo- and hyper-methylated regions (DMRs) in each sex, ranked by methylation change.

**Males**						
**Chromosome**	**Start**	**End**	**Methylation** **Change**	**FDR**	**Gene**	**Genomic Annotation**
13	119,637,001	119,638,000	50.97	0.026	*Ccl28*	Intron
16	30,745,001	30,746,000	42.59	6.93 × 10^−4^	N/A	Intergenic
6	101,083,001	101,084,000	41.81	0.035	N/A	Intron, Intergenic
5	36,531,001	36,532,000	37.56	0.018	*Tbc1d14*	Promoter, 1 to 5 kb, Exon, Intron
8	34,051,001	34,052,000	34.43	0.029	N/A	Intergenic
5	75,512,001	75,513,000	34.13	0.036	N/A	Intergenic
8	14,324,001	14,325,000	32.02	0.001	*Dlgap2*	Intron
5	113,026,001	113,027,000	31.52	0.010	N/A	Intergenic
17	86,302,001	86,303,000	31.15	0.013	*Prkce*	Intron
12	86,540,001	86,541,000	30.63	8.24 × 10^−7^	N/A	Intergenic
19	44,248,001	44,249,000	−71.90	3.34 × 10^−5^	N/A	CpG Island, CpG Shore
1	42,629,001	42,630,000	−56.34	0.010	*Pantr1*	Intron
6	125,711,001	125,712,000	−51.24	0.008	*Ano2*	Promoter, Exon, Intron
11	85,458,001	85,459,000	−47.98	0.022	*Bcas3*	Intron
5	113,462,001	113,463,000	−47.45	6.37 × 10^−7^	N/A	Intron
7	28,925,001	28,926,000	−43.61	0.032	*Actn4*	Intron
5	60,922,001	60,923,000	−41.91	6.44 × 10^−4^	N/A	Intergenic
9	62,371,001	62,372,000	−41.69	0.013	*Anp32a*	1 to 5 kb, Exon, Intron
5	15,589,001	15,590,000	−41.20	0.014	N/A	Intron
18	31,921,001	31,922,000	−40.27	0.007	*Lims2*	Promoter, 1 to 5 kb
**Females**						
**Chromosome**	**Start**	**End**	**Methylation** **Change**	**FDR**	**Gene**	**Genomic Annotation**
10	44,097,001	44,098,000	37.88	0.044	*Crybg1*	Intron
2	153,872,001	153,873,000	37.88	0.007	*Sun5*	Promoter, 1 to 5 kb
2	153,872,001	153,873,000	37.88	0.007	*Bpifb2*	1 to 5 kb
15	73,544,001	73,545,000	35.59	0.027	*Dennd3*	Exon, Intron
12	54,490,001	54,491,000	33.84	0.031	N/A	Intergenic
19	25,422,001	25,423,000	32.99	0.041	*Kank1*	Promoter, Exon, Intron
15	80,000,001	80,001,000	31.60	0.024	*Mir7213*	Promoter, 1 to 5 kb
15	80,000,001	80,001,000	31.60	0.024	*Pdgfb*	Exon, Intron
10	78,470,001	78,471,000	31.09	0.043	N/A	Exon, Intergenic
5	36,308,001	36,309,000	31.04	3.63 × 10^−4^	*Sorcs2*	Intron
6	59,473,001	59,474,000	−37.63	0.002	N/A	Intergenic
2	102,253,001	102,254,000	−37.55	0.022	N/A	Intergenic
7	34,845,001	34,846,000	−33.15	0.037	N/A	Intergenic
11	88,495,001	88,496,000	−30.70	0.035	*Msi2*	Intron
12	40,559,001	40,560,000	−28.42	0.022	*Dock4*	Intron
7	45,521,001	45,522,000	−27.21	0.044	*Plekha4*	1 to 5 kb
7	45,521,001	45,522,000	−27.21	0.044	*Tulp2*	Exon
19	10,904,001	10,905,000	−26.96	0.020	*Prpf19*	Intron
12	102,456,001	102,457,000	−26.28	0.044	N/A	Intergenic, Intron
18	73,806,001	73,807,000	−24.51	0.036	*Me2*	Intron

**Table 5:** DNA methylation changes at DMRs in cardiovascular disease-associated genes that overlap between males and females.

**Males**									
**Gene**	**Chr**	**Start**	**End**	**Lead Meth**	**Ctrl Meth**	**Change in Meth**	**Direc**	**FDR**	**Location**
*Galnt2*	8	124,294,001	124,295,000	30.41	5.38	25.03	Hyper	9.7 × 10^−5^	Promoter, Intron, 1 to 5 kb
		120,995,001	120,996,000	83.38	67.66	15.72	Hyper	0.003	Intron
*Pi16*	17	29,319,001	29,320,000	42.13	15.92	26.22	Hyper	7.8 × 10^−9^	Promoter, 5’UTR, Exon, Intron
*Rbfox1*	16	7,097,001	7,098,000	74.79	95.40	−20.61	Hypo	0.015	Intron
**Females**									
**Gene**	**Chr**	**Start**	**End**	**Lead Meth**	**Ctrl Meth**	**Change in Meth**	**Direc**	**FDR**	**Location**
*Galnt2*	8	121,760,001	121,761,000	75.11	61.03	14.09	Hyper	0.040	Intron
		122,085,001	122,086,000	84.88	70.10	14.78	Hyper	0.003	Intron
*Pi16*	17	29,323,001	29,324,000	64.56	47.71	16.85	Hyper	0.024	Intron
*Rbfox1*	16	6,227,001	6,228,000	49.60	39.36	10.24	Hyper	0.014	Intron

## Data Availability

The ERRBS data presented in this study are openly available in GEO, accession number GSE152711. All other data can be found in the Supplementary Excel file.

## Supplementary Materials

The following are available online at https://www.mdpi.com/1660-4601/18/2/577/s1, Figure S1: Volcano plots depicting DMRs for Pb exposed compared to control in males (**A**) and females (**B**). DMRs in red did not meet the criteria for significance (at least a 10% change in methylation and FDR <0.05). Significantly hypomethylated DMRs are shown in blue, and significantly hypermethylated DMRs are shown in green, Table S1: ERRBS QC data, Table S2: ERRBS QC data, Table S3: DMCs in 5 month female hearts, Table S4: DMRs in 5 month male hearts, Table S5: DMRs in 5 month female hearts, Table S6: Poly-Enrich pathway analysis of hypermethylated DMRs in males, Table S7: Poly-Enrich pathway analysis of hypomethylated DMRs in males, Table S8: Poly-Enrich pathway analysis of hypermethylated DMRs in females, Table S9: Poly-Enrich pathway analysis of hypomethylated DMRs in females, Table S10: Differentially methylated cytosines (DMCs) with direct overlap between male and female hearts, Table S11: Differentially methylated regions (DMRs) with direct overlap between male and female hearts, Table S12: DMC-associated genes that overlap between males and females, Table S13: DMR-associated genes that overlap between males and females.
