# Supplementary figures and images for: Sex-Specific Alterations in Cardiac DNA Methylation in Adult Mice by Perinatal Lead Exposure
Source: Int J Environ Res Public Health. 2021 Jan 12;18(2):577. doi: 10.3390/ijerph18020577 (PMC7826866; doi:10.3390/ijerph18020577)

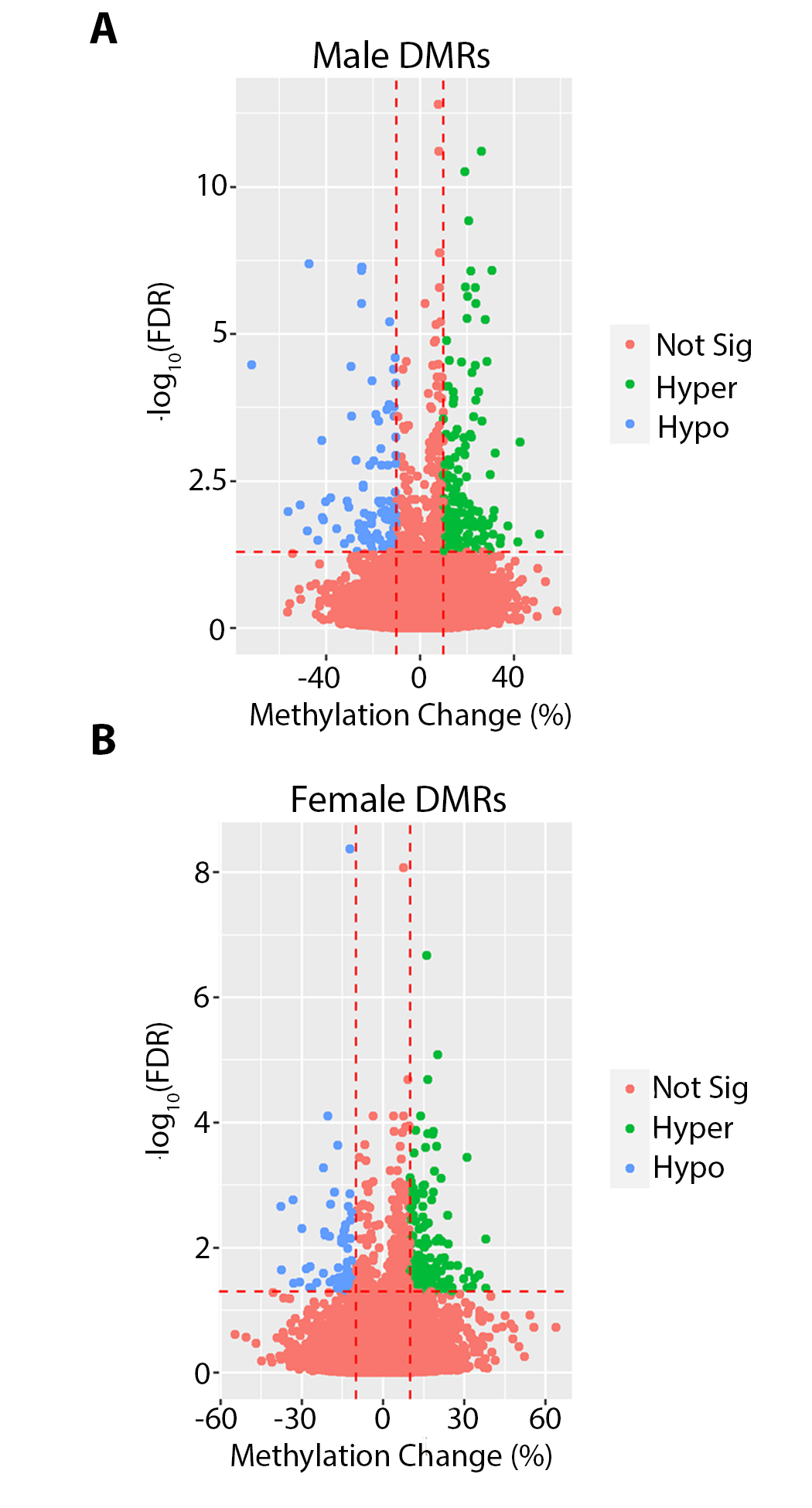

Supplement: Supplementary file 1 [file ijerph-18-00577-s001.zip › Figure S1.tif]

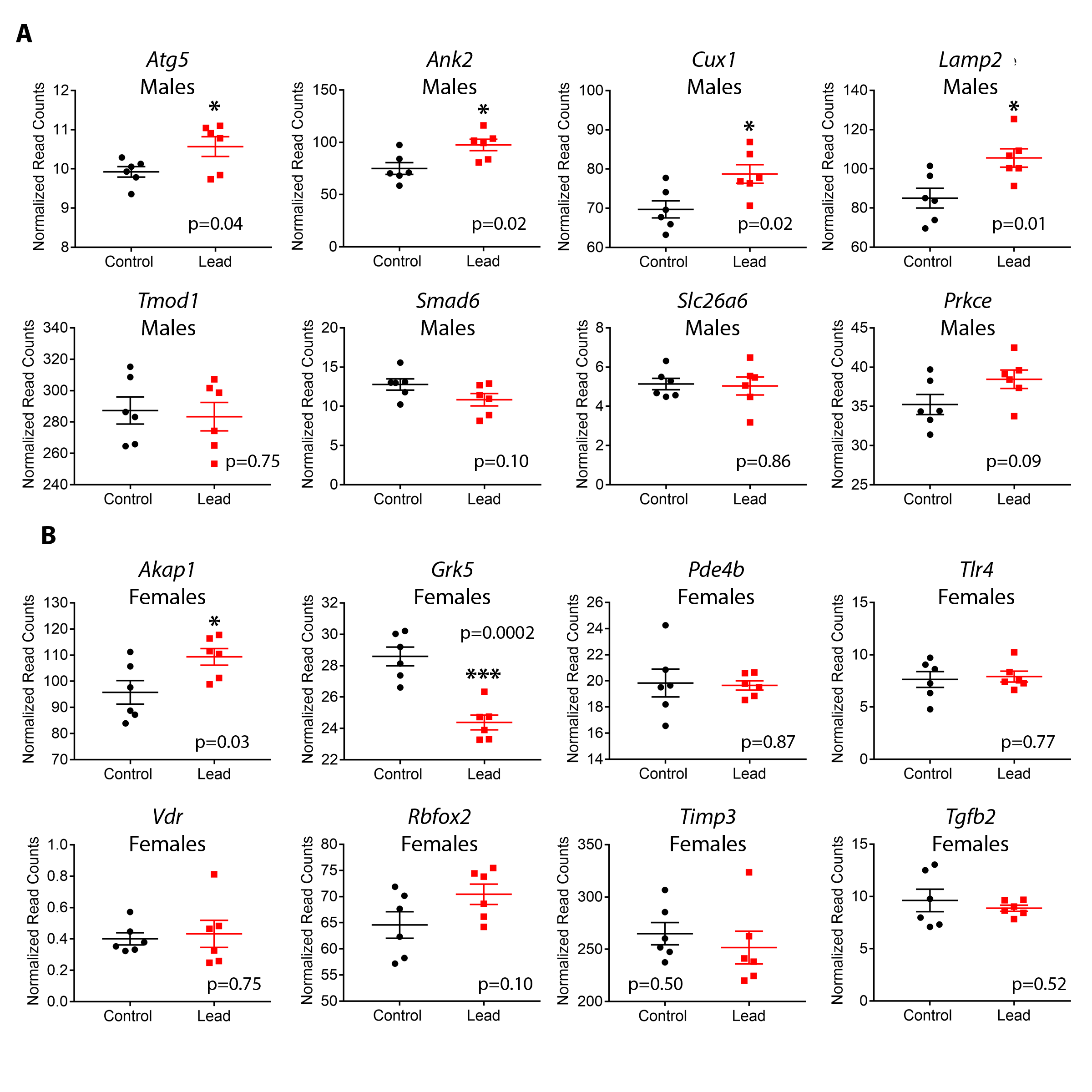

Supplement: Supplementary file 1 [file ijerph-18-00577-s001.zip › Figure S2.tif]
